# Antibiofilm Properties of Antiseptic Agents Used on *Pseudomonas aeruginosa* Isolated from Diabetic Foot Ulcers

**DOI:** 10.3390/ijms231911270

**Published:** 2022-09-24

**Authors:** Koko Barrigah-Benissan, Jerome Ory, Catherine Dunyach-Remy, Cassandra Pouget, Jean-Philippe Lavigne, Albert Sotto

**Affiliations:** 1 Bacterial Virulence and Chronic Infections, INSERM U1047, Department of Microbiology and Hospital Hygiene, CHU Nîmes, Univ Montpellier, Place du Pr Debré, 30029 Nîmes, France; 2 Bacterial Virulence and Chronic Infections, INSERM U1047, Department of Infectious Diseases, CHU Nîmes, Univ Montpellier, Place du Pr Debré, 30029 Nîmes, France

**Keywords:** antibiofilm, Antibiofilmogram^®^, antiseptic, biofilm, chronic wound, *Pseudomonas aeruginosa*, PHMB, PVPI, octenidine, sodium hypochlorite

## Abstract

In diabetic foot ulcers (DFUs), biofilm formation is a major challenge that promotes wound chronicity and delays healing. Antiseptics have been proposed to combat biofilms in the management of DFUs. However, there is limited evidence on the activity of these agents against biofilms, and there are questions as to which agents have the best efficiency. Here, we evaluated the antibiofilm activity of sodium hypochlorite, polyvinylpyrrolidoneIodine (PVPI), polyhexamethylenebiguanide (PHMB) and octenidine against *Pseudomonas aeruginosa* strains using static and dynamic systems in a chronic-wound-like medium (CWM) that mimics the chronic wound environment. Using Antibiofilmogram^®^, a technology assessing the ability of antiseptics to reduce the initial phase of biofilm formation, we observed the significant activity of antiseptics against biofilm formation by *P. aeruginosa* (at 1:40 to 1:8 dilutions). Moreover, 1:100 to 1:3 dilutions of the different antiseptics reduced mature biofilms formed after 72 h by 10-log, although higher concentrations were needed in CWM (1:40 to 1:2). Finally, in the BioFlux200^TM^ model, after biofilm debridement, sodium hypochlorite and PHMB were the most effective antiseptics. In conclusion, our study showed that among the four antiseptics tested, sodium hypochlorite demonstrated the best antibiofilm activity against *P. aeruginosa* biofilms and represents an alternative in the management of DFUs.

## 1. Introduction

Diabetic foot ulcers (DFUs) in people with diabetes mellitus are a major public health problem. Its prevalence is high, affecting 15–25% of patients with diabetes at least once in their lifetime [1,2]. An infected DFU is a major complication; it is the main reason for diabetes-related hospitalizations and a major cause of amputation [1,3]. DFUs are frequently colonized and then infected by microorganisms organized in biofilms inside the wound bed [4,5,6]. The biofilm is a structure of microorganism aggregates that participates in the chronicity of the lesion and delays wound healing [6]. The production of an extracellular polymeric substance (EPS) matrix [7] by microorganisms is essential for their adaptation to nutritional and environmental conditions [8]. It also facilitates bacterial protection against the hostile environment [6].

*Pseudomonas aeruginosa* is a ubiquitous and opportunistic pathogen with a prominent role in many infectious diseases, such as nosocomial urinary tract infections, cystic fibrosis infections or chronic wounds, such as DFU infections [9]. In DFUs, this microorganism is particularly prevalent [10], notably in warm countries, and plays a key role in biofilm organization [9,11,12]. The management of biofilms on DFUs is essential [13] and consists of eliminating the biofilm and disrupting the structural stability of the EPS through extensive debridement [14]. However, debridement is not sufficient to remove all of the microorganisms present in the wound bed [15,16]. Several additional antibiofilm strategies have been developed to complement the classical management of DFUs to eradicate bacterial biofilms and improve the healing process [17].

Antiseptics are one of the antimicrobial agents that can be used in the antibiofilm DFU strategy due to their anti-infective benefits [13,18,19,20]. Although the International Working Group on the Diabetic Foot (IWGDF) recommends against using topical antiseptics in DFUs [1], there is evidence that some antiseptics reduce the microbial load of the wounds [21,22]. This reinforces the need for studies to evaluate the value of antiseptics in chronic wounds.

Many biofilm models have been developed to study antimicrobial susceptibility (e.g., in vitro static, in vitro dynamic or even in vivo models) [23,24]. However, none are suitable for evaluating this activity while taking into account the wound microenvironment [23,25]. Most in vitro models generally lack the interaction of the biofilm with the host cells and are not representative of the wound microenvironment [24], whereas in vivo models are subject to inter-individual variation, are expensive and also fail to reproduce the wound microenvironment [24,25]. Recently, we developed a new in vitro medium, the Chronic Wound Medium (CWM), that mimics the microenvironment encountered in chronic wounds [17]. Using this medium, *P. aeruginosa* formed a static biofilm faster and decreased its virulence in a *Caenorhabditis elegans* model after culture in CWM [26]. Moreover, we integrated this medium into a microfluidic system, the BioFlux^TM^ 200, a new and emerging technology adapted to study dynamic biofilm formation and the antibiofilm effects of different compounds [26].

Herein, we assessed the antibiofilm properties of four antiseptic agents (sodium hypochlorite, polyvinylpyrrolidone iodine (PVPI), polyhexamethylene biguanide (PHMB) and octenidine) on clinical *P. aeruginosa* strains isolated from infected DFUs using two in vitro static models and one dynamic model.

## 2. Results

### 2.1. Evaluation of the Susceptibility of P. aeruginosa Strains to Antiseptics

We evaluated the susceptibility of four strains of *P. aeruginosa* (PAO1 and three clinical strains, PAC1, PAC2 and PAC4) to four compounds (sodium hypochlorite, PHMB, PVPI and octenidine). The MIC and partial biofilm Minimum Inhibitory Concentration (MICb) values of the antiseptics are displayed with their concentrations (10^−3^ g·L^−1^) and ratios in commercial solutions (V:V) (Table 1). MIC and MICb values were obtained in six replicates for each antiseptic agent for each strain. The MIC values were 1660 × 10^−3^ g·L^−1^ (1:3 dilution factor) for sodium hypochlorite, 12,500 × 10^−3^ g·L^−1^ (1:8 dilution factor) for PVPI, 15.6 × 10^−3^ g·L^−1^ (1:64 dilution factor) for PHMB and 7.8 × 10^−3^ g·L^−1^ (1:64 dilution factor) for octenidine. All MIC values were lower than the commercial concentrations, regardless of the antiseptic agent. The same trend was observed for MICb values (625 × 10^−3^ g·L^−1^ (1:8 dilution factor) for sodium hypochlorite; 5000 × 10^−3^ g·L^−1^ (1:20 dilution factor) for PVPI; 25 × 10^−3^ g·L^−1^ (1:40 dilution factor) for PHMB; and 12.5 × 10^−3^ g·L^−1^ (1:40 dilution factor) for octenidine).

Two antiseptic profiles were observed. The first profile, including sodium hypochlorite and PVPI, had MICb values that were lower than the MIC values, whereas the second one, including PHMB and octenidine, presented MICb values higher than the MIC values.

### 2.2. Effects of Antiseptics on the Biofilm Living Bacterial Load

**Figure 1:**
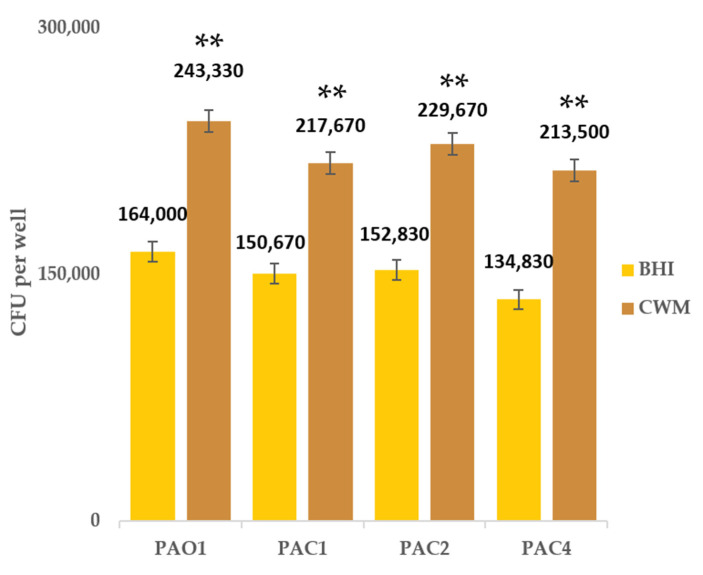
Mean living bacterial load after 72 h biofilm formation by *P. aeruginosa* strains (the reference strain PAO1 and three clinical strains, PAC1, PAC2 and PAC4) cultivated in two media, BHI and CWM. The average CFU per well was calculated from six independent experiments and determined by an automatic counting system. Results are presented as the mean ± standard deviation. Statistics were performed using a Wilcoxon Rank-Sum test using the R^®^ software in its 4.0.2 version to compare the difference in the mean bacterial load of each strain in two culture media. **, *p* < 0.01.

We first quantified the bacterial loads of mature *P. aeruginosa* biofilms at 72 h [27]. The strains were cultivated in the reference medium (BHI) and CWM. The results are presented in Figure 1 and Table S1 (see supplementary materials). No statistical difference in the mean living bacterial load was noted when the bacteria were cultivated in the BHI medium (*p* = not significant, NS). Similarly, no difference was observed when bacteria were cultivated in CWM (*p* = NS). Interestingly, the mean load value distribution was significantly higher when *P. aeruginosa* strains were cultivated in CWM compared to BHI (*p* < 0.01), demonstrating that CWM conditions favor biofilm formation.

We next evaluated the activity of antiseptic agents on the living bacterial load (Table 2). In practice, antiseptic agents are used to complement the bacterial offloading action of debridement by 10 to 15% [15]. We then determined the antiseptic concentrations needed to obtain a 10-log reduction in the living bacterial load of the studied *P. aeruginosa* strains. The same sodium hypochlorite concentration was needed, regardless of the studied strain and the culture medium used. This concentration was equivalent to the MIC previously determined. For the three other antiseptic agents, higher concentrations were needed to obtain a 10-log reduction in the living bacterial load in CWM compared to BHI. No variations were noted between strains, except for PAC4, for which the concentrations of PHMB and octenidine were higher than those used for the other strains. Interestingly, the concentration of PHMB was lower than the MIC values obtained with the studied strains, whereas the concentration of octenidine was higher.

### 2.3. Antiseptic Efficiency after In Vitro Automatized Debridement on a Pre-formed P. aeruginosa Biofilm

#### 2.3.1. Evaluation of *P. aeruginosa* Biofilm Formation under Flow Conditions

To evaluate the potential of biofilm formation by the four strains, we determined the percentage of biofilm formed in the BioFlux^TM^ 200 system in the control BHI medium and in CMW [26].

All strains were able to form biofilms and to remain attached under shear force (Table 3). The kinetics of biofilm formation of the reference strain PAO1 showed no difference in the percentage of biofilm formation after 24 h between the two media (*p* = NS), whereas these percentages were significantly different at 48 h (*p* < 0.1) and at 72 h (*p* < 0.01). All three clinical strains demonstrated significantly higher percentages of biofilm formation when bacteria were cultivated in CWM compared to BHI, regardless of the time point (Table 3).

#### 2.3.2. Antiseptic Efficiency on a Pre-formed *P. aeruginosa* Biofilm under Flow Conditions

**Figure 2:**
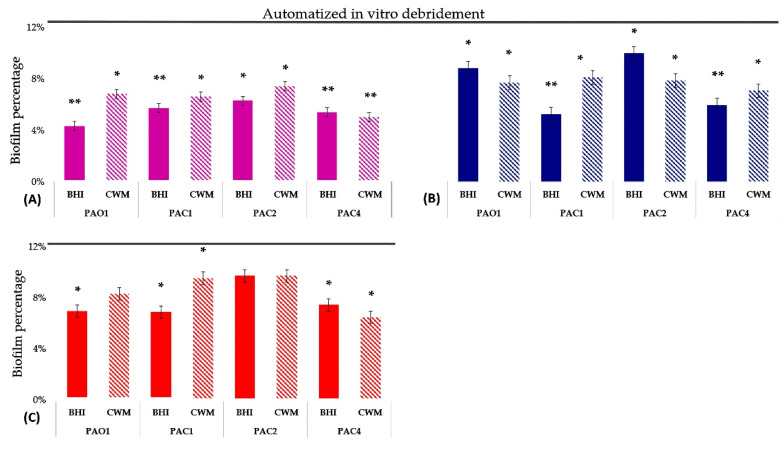
Percentage of biofilm reduction in pre-formed *P. aeruginosa* biofilms using commercialized concentrations of three antiseptics (sodium hypochlorite (**A**, pink), PHMB (**B**, blue) and octenidine (**C**, red)) in BHI and CWM. In vitro automatized debridement left a mean of 12% of biofilm in the microfluidic channel before antiseptic treatment after 72 h of culture. The mean percentage of biofilm after in vitro automatized debridement and before antiseptic treatment is represented by the dashed line. Samples were tested in six independent experiments. Results are presented as the mean ± standard deviation. Statistics were performed using a t-test using the R^®^ software version 4.0.2 to compare the efficiency of antiseptics on biofilms after in vitro automatized debridement. *, *p* < 0.1; ** *p* < 0.01.

To mimic the management of chronic wounds and evaluate antiseptic action in a constituted biofilm, we developed a method for the in vitro automatized debridement of the biofilm inside the BioFlux^TM^ system, as previously validated [26]. After in vitro automatized debridement, we administered the four antiseptic agents at the commercialized concentrations on pre-formed biofilms of all strains in BHI and CWM. The results are presented in Figure 2 and detailed in Table S2 (Supplementary Data).

After in vitro automatized debridement of the biofilm formed inside the BioFlux^TM^ system, a mean of 12% (see Supplementary Data, Table S2) of bacteria remained in biofilms and were not completely removed to mimic clinical data that estimated the effectiveness of debridement at around 85–90% [16]. We administered the antiseptics at commercial concentrations without dilution. We were unable to test the activity of PVPI, as its color interfered with the MetaVue^TM^ software (Molecular Devices, Sunnyvale, CA, USA).

The use of sodium hypochlorite and PHMB significantly reduced the pre-formed biofilm, regardless of the strain and the medium used (*p* < 0.01) (Figure 2). The residual biofilm percentage was reduced to 5.98% in BHI and 6.8% in CWM for sodium hypochlorite and 8.5% in BHI and 8.54% in CWM for PHMB (see Supplementary Data, Table S2). However, the antiseptic efficiency was better when bacteria were cultivated in BHI than in CWM. The results were further improved when we evaluated the octenidine action, which showed strain-dependent efficiency. The use of this antiseptic significantly reduced the pre-formed biofilms of PAO1, PAC1 and PAC4 cultivated in BHI and PAC1 and PAC4 cultivated in CWM (*p* < 0.01). However, no significant effects were observed when octenidine was administered after the in vitro automatized debridement of the biofilm formed by the PAC2 strain (in CWM and BHI) and PAO1 (in CWM only) (*p* = NS).

## 3. Discussion

The diagnosis of chronic wound infections remains problematic, leading to the inappropriate use of antibiotics and a high prevalence of multidrug-resistant bacteria carriage [28]. Moreover, commensal and pathogenic bacteria are organized in biofilms, maintaining the chronicity of the lesion and delaying wound healing [7]. New alternative solutions must be developed and evaluated. Our study aimed to assess the antibiofilm properties of four antiseptic agents (sodium hypochlorite, PVPI, PHMB and octenidine) against *P. aeruginosa* strains, a species frequently isolated in chronic wounds [10] and classified as a strong biofilm producer [29,30]. Moreover, we studied this activity in a medium mimicking the environment encountered in chronic wounds to better evaluate the antiseptic effects.

The MIC values of the four antiseptic agents against our panel of four *P. aeruginosa* strains were always lower than initial commercialized concentrations. Previous studies have evaluated the MIC values of antiseptics on *P. aeruginosa* strains (see Supplementary Data, Table S3).

For sodium hypochlorite, the MIC values obtained in our study (1666 mg·L^−1^ and 1:3 dilution) are 3- to 5-fold higher than the ones previously obtained [31,32,33,34,35]. This variation may be related to the differences in the studied strains and in the media used, as previously noted [36]. We observed the same trend using PVPI. The MIC was 12,500 mg·L^−1^ (1:8 dilution factor), higher than the values found in previous studies [37,38,39,40]. However, although the values obtained were high, their dilution ratios were low, varying between 0.04% and 5% (1:2 to 1:250 dilution factor) [37,38,39,40]. Moreover, we noted no difference in the MIC values of the other antiseptics compared with previous studies, suggesting that the BHI medium was not solely responsible for the results obtained with the two first antiseptics. The MIC values of PHMB and octenidine were 15.6 mg·L^−1^ and 7.8 mg·L^−1^, respectively, in accordance with previous studies [33,37,40,41,42,43]. These findings support weaker concentrations compared to the commercialized solutions routinely used.

Classically, most studies on antiseptic efficiency determined the Minimal Biofilm Eradication Concentration (MBEC) values to evaluate the potential of antiseptics to eradicate mature and established biofilms. Here, we adapted the Antibiofilmogram^®^ test to obtain the MICb and assess the antiseptic impact on the biofilm formation kinetics [44,45]. The principle was to use a diagnostic tool derived from the BioFilm Ring Test^®^ (BioFilm Control, St Beauzire, France). This test studies early biofilm formation by bacteria. We adapted this method to assess the ability of antiseptics to inhibit early biofilm formation [44]. We determined the MICb, reflecting the minimum concentration of antiseptic agents that inhibits biofilm formation. Our MICb values were lower than commercial concentrations, with dilution ratios ranging from 1:8 for sodium hypochlorite to 1:40 for PHMB and octenidine. The comparison between MICb and MIC values revealed two profiles of antiseptic agents: one profile (for sodium hypochlorite and PVPI) with MICb values higher than MIC values, suggesting that biofilm formation was inhibited at lower concentrations than those needed to inhibit bacterial growth. The second profile (for PHMB and octenidine) had MICb values lower than MIC values, suggesting that biofilm formation was inhibited by concentrations higher than those needed to inhibit bacterial growth, and a delayed antibiofilm formation effect. This effect is certainly related to faster biofilm formation upon exposure to antiseptic agents rather than a defense mechanism. Thus, higher octenidine and PHMB concentrations are needed to inhibit biofilm formation.

Our results thus highlighted two profiles of the studied antiseptic agents. Sodium hypochlorite and PVPI can complement wound debridement by preventing new biofilm formation without altering bacterial growth. PHMB and octenidine can also be interesting in complementing debridement procedures by coupling antibiofilm formation properties with bacterial growth inhibition properties. For both profiles, at commercial concentrations, all tested antiseptics induced bacterial growth inhibition and biofilm formation inhibition. These findings shine a light on the antimicrobial effects of the antiseptic agents in the early phases of biofilm formation.

We also assessed the effect of the selected antiseptics on the living bacterial load of mature biofilms, which are predominant in chronic wounds [7]. First, we observed that the biofilms formed after 72 h were denser and heavier, and the bacterial load was higher when *P. aeruginosa* strains were cultivated in CWM compared to BHI, as previously reported [26]. This effect was confirmed in the dynamic flow model, BioFlux^TM^ 200, where we also noted the influence of CWM on the development of biofilm formation. Altogether, we demonstrated the influence of the environment on biofilm formation. The decreased antimicrobial activity of antiseptics has been documented in the presence of organic matter, such as the blood included in CWM [46,47]. Due to the denser biofilm matrix and the effect of the medium mimicking the wound environment, a 10-log higher antiseptic concentration (PVPI, PHMB and octenidine) was necessary to reduce the living bacterial load. Sodium hypochlorite was an exception, with no difference in the MICs needed to obtain this 10-log reduction in biofilms of *P. aeruginosa* cultivated in BHI or CWM. This result could be related to the fact that this antiseptic agent is less affected by the organic load [48].

In a biofilm, antiseptic penetration is reduced with a decreased growth rate of persistent cells [49]. We thus developed a protocol to test the efficiency of antiseptics after in vitro automatized debridement, mimicking a key step in the management of chronic wounds [26]. We observed the efficiency of sodium hypochlorite and PHMB and the strain-dependent activity of octenidine. In clinical practice, 85% of biofilm bacterial load offloading is obtained through debridement, with 10 to 15% of residual biofilm. Our results indicate that sodium hypochlorite can significantly complement wound debridement and reduce this percentage to around 6%, and PHMB can reduce it to 8%. This residual biofilm allows residual microbiota, which participates in DFU healing [50,51]. In addition, this biofilm reduction was effective 72 h after the antiseptic addition. Previous studies demonstrated that adhesion to surfaces for biofilm formation was accelerated in *P. aeruginosa* following their release from biofilms, which happens through debridement [52]. This study highlighted that sodium hypochlorite and PHMB prevented this faster biofilm formation. These results could be due to the mechanism of action of the antiseptic agents. Indeed, for octenidine, this mechanism is directly linked to its interaction with the bacterial membrane [53]. It is possible that the EPS plays a role in biofilm survival and reduces the ability of octenidine to reach the membrane of *P. aeruginosa*. Octenidine activity was particularly reduced when the antiseptic was used against bacteria cultivated in CWM, which enhanced the biofilm density and thickness. Moreover, *P. aeruginosa* was previously reported to adapt to octenidine by membrane remodeling and the production of efflux pumps [54]. Investigating these mechanisms in biofilm models would be a fruitful area for further work. Although the PHMB effect was also linked to its interaction with the bacterial membrane [55], the commercial solution used in our study also included betaine, a compound that disrupts EPS proteins [56], explaining the antibiofilm properties of PHMB. Finally, sodium hypochlorite has multiple mechanisms of action (reactive oxygen species, protein degradation and oxidizing action) with passive diffusion inside the microorganisms [48], suggesting that this antiseptic is an optimal candidate for eliminating bacterial biofilms associated with chronic wounds. Overall, our results reinforce the need to use a dynamic in vitro system and media mimicking the conditions encountered in the clinical situation.

## 4. Materials and Methods

### 4.1. Bacterial Strains, Culture Conditions and Antiseptic Agents

The bacteria and media used in this study are listed in Table 4

CWM is a previously described and patented in vitro media model that reproduces the chronic wound environment [17,26]. CWM has a fixed pH of 8, as seen in non-healing wounds [26]. Its composition includes Bolton broth, heat-inactivated human serum, debris of human keratinocytes and hemolyzed human blood.

Four previously characterized *P. aeruginosa* strains were used. PAO1, a reference strain [57,58], and three clinical strains (PAC1 [17], PAC2 [52] and PAC4 [52]) isolated from Grade 3 [59] diabetic foot ulcers of patients at the University Hospital of Nîmes.

Bacteria were grown overnight in bacterial culture tubes with shaking at 200 rpm in aerobic conditions at 37 °C in brain heart infusion (BHI, Sigma-Aldrich, Saint-Quentin-Fallavier, France) broth or CWM (European patent application EP21305337) [17].

Four antiseptic agents were tested for tolerance and available data on their in vitro or clinical efficiency on chronic wounds: (i) sodium hypochlorite, 5 g·L^−1^ (Dakin^®^, Cooper, France), (ii) PVPI or Povidone iodine, 100 g·L^−1^ (Betadine^®^, Mylan Medical, France), (iii) PHMB, 1 g·L^−1^ (Prontosan^®^ 0.1% undecylenamidopropyl-betaine and 0.1% PHMB in aqueous solution; B. Braun Melsungen AG) and (iv) octenidine 0.5 g·L^−1^ (Octenelin^®^ wound irrigation solution, Schuelke und Mayr, Norderstedt, Germany).

### 4.2. Susceptibility Testing Assays

Antimicrobial susceptibility testing (MIC) of isolates was performed by the broth microdilution method on BHI according to EUCAST recommendations (https://www.eucast.org/clinical_breakpoints, accessed on 2 August 2022). Fresh bacterial cultures were used after overnight incubation at 37 °C in aerobic conditions. The range of dilution was 1:2 to 1:2048 for all antiseptic agents. Each antiseptic agent was tested in six independent assays in six replicates for each strain.

Antibiofilmogram^®^ [44,60] is a diagnostic tool using the Biofilm Ring Test^®^ to study antiseptic actions on biofilm formation [61]. Briefly, *P. aeruginosa* strains were subcultured on BHI agar at 37 °C for 24 h. Six colonies were inoculated into BHI broth and homogenized. The bacterial suspension was standardized to an optical density (OD) of 1.00 ± 0.05 at 600 nm and diluted 1:250 in BHI broth to obtain a final concentration of 4 × 10^6^ CFU/mL using a defined calibration curve between OD and CFU/mL. Two hundred microliters of this bacterial suspension, 1% (vol/vol) magnetic beads (TON004) and antiseptics (20 μL of dilutions of each antiseptic) were then incubated, in triplicate, at 37 °C for 4 h (time needed to form *P. aeruginosa* biofilm in this model [17]) in a 96-well microplate (Falcon 96 Flat Bottom Transparent, Corning, United States). The microplate was placed onto a magnetic block for 1 min and analyzed. Due to the color of the antiseptic agent solutions, it was not possible to use a microplate scanner, and we compared the results visually. The MICb was therefore defined as the minimum concentration of the antiseptic agent that inhibited early biofilm formation. This MICb was visually detected as the minimum concentration that allowed brown spot formation at the bottom of the wells. Assays were replicated six times. Four wells without antiseptics filled with the bacterial suspension and magnetic beads were used as the positive control.

### 4.3. Antiseptic Effect on 72 h Biofilm Living Bacterial Load

The living bacterial load of 72 h-old biofilms before and after the antiseptic agent addition was quantified using an adapted method previously described [27]. Fresh overnight bacterial cultures were adjusted to an OD_600 nm_ of 1 ± 0.05. The cultures were then diluted at 1:100 and incubated for 72 h at 37 °C without shaking in three flat-bottom 96-well plates for biofilm formation. Fresh culture medium was added every 24 h until 72 h. Wells with medium without bacterial culture were used as the negative control. At 72 h, one plate was used as the control for biofilm formation (C1). In the second plate, several dilutions (1:2 to 1:1028) of each antiseptic agent were added in three replicates. The plates were then incubated for an additional 24 h at 37 °C. The third plate served as a negative antiseptic effect control (C2), containing only fresh culture medium incubated for an additional 24 h at 37 °C. After incubation, the microplates were washed three times with 200 μL of 1× PBS. Finally, 200 μL of 1× PBS was added to the well before biofilm disruption by sonication for 10 min at 40 kHz. Each well was then serially diluted, and the last dilution was plated on LB agar. The agar plates were then incubated overnight at 37 °C, and CFUs were counted to quantify the living bacterial load in each well. We determined the concentration of each antiseptic needed to obtain a 10-log reduction in the living bacterial load, since antiseptic agents are used to complement debridement offloading by 10 to 15%. The experiment was performed six times for each strain.

### 4.4. Dynamics of Antiseptic Agents on Biofilm

The previously described BioFlux^TM^ 200 microfluidic system (Fluxion Biosciences Inc., Alameda, CA, USA) was adapted to assess the dynamics of biofilm formation and biofilm quantification, along with the dynamics of antiseptic agents in biofilm disruption [62]. This system is composed of microfluidic channels incorporated in 48-well plates. The bottom of the plate is made of a 180 µm glass coverslip, allowing microscopic examination [63]. Thus, the system was coupled with a Leica DM IRB inverted fluorescence microscope for biofilm visualization and a CoolSNAP FX black and white camera (Roper Scientific^®^, Trenton NJ, United States) for automated image acquisition and recording. Images were processed with MetaVue^TM^ software (Molecular Devices, Sunnyscale, CA, USA). Image analysis was processed using ImageJ^®^ software. The analysis included 16-bit grayscale image adjustment with the threshold function, and the biofilm percentage was calculated with the “Analyzes particles” function, as previously established [62,64]. Colonies of *P. aeruginosa* strains were resuspended in 3 mL of BHI and were incubated at 37 °C with shaking (220 rpm) overnight. A bacterial suspension was then prepared from this overnight culture standardized to an OD_600_ of 0.1 ± 0.05 following a serial 1:200 dilution. The channel was first primed with 500 µL of medium without bacteria in the inflow well with a pressure setting of 1 dyne/cm^2^ for 10 min. The remaining medium in the well was then withdrawn. The microfluidic channels were then inoculated by injecting the bacterial suspension from the output reservoir for 30 min at 1 dyne/cm^2^. The setup was placed on the heating plate at 37 °C. Finally, the bacterial suspension was added to the inflow well for 72 h with pressure and temperature settings of 0.2 dyne/cm^2^ and 37 °C. Fresh medium was added every 24 h to the input well, and the medium in the output well was discarded. Biofilms were recorded at 24, 48 and 72 h.

To mimic the first step of clinical management of a chronic wound, we developed an in vitro automatized debridement method to remove part of the pre-formed biofilm [26]. After 72 h of biofilm formation, a shear flow of 5 dynes/cm^2^ was applied from the inflow well to the outflow well for 10 min. The flow was then reversed between the inflow and the outflow wells for 10 min. This process of flow reversion was performed twice. The aim of this flow inversion was to significantly reduce the biofilm constituted in the microfluidic channel to 10–15% of the remaining biofilm in the channel to mimic in vivo conditions [16]. After this in vitro automatized debridement, a dynamic flow (0.2 dyne/cm^2^ at 37 °C for 24 h) of antiseptics was applied to evaluate their actions on the remaining pre-formed biofilm. The residual biofilm percentage after antiseptic contact was calculated by dividing the biofilm percentage after antiseptic contact by the biofilm percentage before antiseptic contact *100. The concentrations of antiseptics used corresponded to the commercialized compounds at their pure concentrations.

### 4.5. Statistical Analysis

All statistical analyses were carried out using the R^®^ software version 4.0.2. A Wilcoxon Rank-Sum test was used to analyze the distribution of the living bacterial load results and assess the residual biofilm in the dynamic model.

## 5. Conclusions

In chronic wounds, pathological biofilms formed by *P. aeruginosa* are particularly prevalent and difficult to treat. Indeed, new therapeutic strategies are needed, and the determination of the in vitro efficiency against pre-formed biofilms is a great first step in the discovery of methods for the future management of these infections. Our study highlighted that sodium hypochlorite was the most effective antiseptic against biofilms formed by *P. aeruginosa*. This compound was active against these bacteria at concentrations lower than the commercialized one. It presented a MICb lower than MIC (1:8 vs. 1:3), demonstrating its activity against biofilm formation. Moreover, at this concentration, it reduced the living *P. aeruginosa* load formed in biofilms by 10-log, suggesting that there was no need to increase the antiseptic concentration to reduce mature biofilms. Finally, the use of sodium hypochlorite significantly reduced the biofilms after in vitro automatized debridement. Clinical studies are now necessary to confirm the results of this study, demonstrating that antiseptics are an interesting alternative solution for the management of DFUs.

## Figures and Tables

**Table 1:** Minimum Inhibitory Concentration (MIC) and partial biofilm Minimum Inhibitory Concentration (MICb) values (in 10^−3^ g·L^−1^) and dilution ratios (in V:V) determined for the reference strain PAO1 and three clinical *P. aeruginosa* strains (PAC1, PAC2 and PAC4) against four antiseptic solutions.

	Sodium Hypochlorite				PVPI				PHMB				Octenidine			
	MIC	V:V	MICb	V:V	MIC	V:V	MICb	V:V	MIC	V:V	MICb	V:V	MIC	V:V	MICb	V:V
PAO1	1660	1:3	625	1:8	12,500	1:8	5000	1:20	15.6	1:64	25	1:40	7.8	1:64	12.5	1:40
PAC1	1660	1:3	625	1:8	12,500	1:8	5000	1:20	15.6	1:64	25	1:40	7.8	1:64	12.5	1:40
PAC2	1660	1:3	625	1:8	12,500	1:8	5000	1:20	15.6	1:64	25	1:40	7.8	1:64	12.5	1:40
PAC4	1660	1:3	625	1:8	12,500	1:8	5000	1:20	15.6	1:64	25	1:40	7.8	1:64	12.5	1:40

**Table 2:** Antiseptic concentrations (in 10^−3^ g·L^−1^) and dilution ratios (in V:V) needed to obtain a 10-log reduction in the living bacterial load of *P. aeruginosa* strains (the reference strain PAO1 and three clinical strains, PAC1, PAC2 and PAC4) cultivated in two media, BHI and CWM.

	Sodium Hypochlorite				PVPI				PHMB				Octenidine			
	BHI	V:V	CWM	V:V	BHI	V:V	CWM	V:V	BHI	V:V	CWM	V:V	BHI	V:V	CWM	V:V
PAO1	1660	1:3	1660	1:3	12,500	1:8	50,000	1:2	10	1:100	20	1:50	10	1:50	12.5	1:40
PAC1	1660	1:3	1660	1:3	12,500	1:8	50,000	1:2	10	1:100	20	1:50	10	1:50	12.5	1:40
PAC2	1660	1:3	1660	1:3	12,500	1:8	50,000	1:2	10	1:100	20	1:50	10	1:50	12.5	1:40
PAC4	1660	1:3	1660	1:3	12,500	1:8	50,000	1:2	10	1:100	25	1:40	10	1:50	25	1:20

**Table 3:** Kinetics of biofilm formation by *P. aeruginosa* strains in the BioFlux^TM^ system. The percentages of biofilm formation were determined for the reference strain PAO1 and three clinical strains, PAC2, PAC3 and PAC4, at 24 h, 48 h and 72 h post-incubation in BHI and CWM after six independent experiments. Results are presented as the mean ± standard deviation. Statistics were performed using a *t*-test in GraphPad Prism version 9.2 to compare the percentage of biofilm formed in BHI and CWM for each time point.

	Percentages of Biofilm Formation								
	At 24 h			At 48 h			At 72 h		
	BHI	CWM	*p*	BHI	CWM	*p*	BHI	CWM	*p*
PAO1	12% ± 0.3	13% ± 0.5	NS	38% ± 0.1	53% ± 0.3	<0.1	70% ± 0.1	99% ± 0.2	<0.01
PAC1	11% ± 0.4	25% ± 0.5	<0.01	45% ± 0.5	64% ± 0.3	<0.01	69% ± 0.5	99% ± 0.2	<0.01
PAC2	14% ± 0.5	17% ± 0.3	<0.1	42% ± 0.4	59% ± 0.5	<0.01	70% ± 0.2	98% ± 0.2	<0.01
PAC4	12% ± 0.3	15% ± 0.2	<0.1	42% ± 0.5	62% ± 0.2	<0.01	75% ± 0.5	99% ± 0.1	<0.01

**Table 4:** Bacterial strains and media used in this study.

Strain	Characteristics	References
PAO1	Reference strain	[57,58]
PAC 1	*Pseudomonas aeruginosa* clinical strain isolated from a Grade 3 DFI * (patient n°1)	[17]
PAC 2	*Pseudomonas aeruginosa* clinical strain isolated from a Grade 3 DFI * (patient n°2)	[52]
PAC 4	*Pseudomonas aeruginosa* clinical strain isolated from a Grade 3 DFI * (patient n°3)	[52]
Media		
BHI	Brain heart infusion	Sigma-Aldrich
LB	Luria–Bertani broth	Sigma-Aldrich
CWM	Patented medium mimicking in vivo conditions encountered in chronic wounds	[17,26]

* DFI, diabetic foot infection.

## Data Availability

Data supporting the reported results can be found in the Bioflux software at the U1047 unit in the file: Chronic wound research section.

## Supplementary Materials

The following supporting information can be downloaded at: https://www.mdpi.com/article/10.3390/ijms231911270/s1.

## References

[1] Lipsky B.A., Senneville É., Abbas Z.G., Aragón-Sánchez J., Diggle M., Embil J.M., Kono S., Lavery L.A., Malone M., van Asten S.A. (2020). Guidelines on the diagnosis and treatment of foot infection in persons with diabetes (IWGDF 2019 update). Diabetes Metab. Res. Rev..

[2] Foot O., Clinic A. Diabetic Foot: Facts and Figures koo. DF Blog 2015. https://diabeticfootonline.com/diabetic-foot-facts-and-figures/.

[3] Fosse-Edorh S., Mandereau-Bruno L., Regnault N. (2015). Le poids des complications liées au diabète en France en 2013. Synthèse et perspectives. BEH.

[4] Malone M., Bjarnsholt T., McBain A.J., James G.A., Stoodley P., Leaper D., Tachi M., Schultz G., Swanson T., Wolcott R.D. (2017). The prevalence of biofilms in chronic wounds: A systematic review and meta-analysis of published data. J. Wound Care.

[5] Metcalf D.G., Bowler P.G. (2016). Clinician perceptions of wound biofilm. Int. Wound J..

[6] Pouget C., Dunyach-Remy C., Pantel A., Schuldiner S., Sotto A., Lavigne J.-P. (2020). Biofilms in Diabetic Foot Ulcers: Significance and Clinical Relevance. Microorganisms.

[7] Percival S.L., McCarty S.M., Lipsky B. (2015). Biofilms and Wounds: An Overview of the Evidence. Adv. Wound Care.

[8] Bjarnsholt T. (2013). The role of bacterial biofilms in chronic infections. APMIS.

[9] Mulcahy L.R., Isabella V.M., Lewis K. (2014). *Pseudomonas aeruginosa* biofilms in disease. Microb. Ecol..

[10] Serra R., Grande R., Butrico L., Rossi A., Settimio U.F., Caroleo B., Amato B., Gallelli L., de Franciscis S. (2015). Chronic wound infections: The role of *Pseudomonas aeruginosa* and *Staphylococcus aureus*. Expert Rev. Anti Infect. Ther..

[11] Rahim K., Saleha S., Zhu X., Huo L., Basit A., Franco O.L. (2017). Bacterial Contribution in Chronicity of Wounds. Microb. Ecol..

[12] Fazli M., Bjarnsholt T., Kirketerp-Møller K., Jørgensen B., Andersen A.S., Krogfelt K.A., Givskov M., Tolker-Nielsen T. (2009). Nonrandom Distribution of *Pseudomonas aeruginosa* and *Staphylococcus aureus* in Chronic Wounds. J. Clin. Microbiol..

[13] Schultz G., Bjarnsholt T., James G.A., Leaper D.J., McBain A.J., Malone M., Stoodley P., Swanson T., Tachi M., Wolcott R.D. (2017). Consensus guidelines for the identification and treatment of biofilms in chronic nonhealing wounds. Wound Repair Regen..

[14] Sibbald R.G., Elliott J.A., Persaud-Jaimangal R., Goodman L., Armstrong D.G., Harley C., Coelho S., Xi N., Evans R., Mayer D.O. (2021). Wound Bed Preparation 2021. Adv. Ski. Wound Care.

[15] Schwartz J.A., Goss S.G., Facchin F., Avdagic E., Lantis J.C. (2014). Surgical debridement alone does not adequately reduce planktonic bioburden in chronic lower extremity wounds. J. Wound Care.

[16] Mori Y., Nakagami G., Kitamura A., Minematsu T., Kinoshita M., Suga H., Kurita M., Hayashi C., Kawasaki A., Sanada H. (2019). Effectiveness of biofilm-based wound care system on wound healing in chronic wounds. Wound Repair Regen..

[17] Pouget C., Dunyach-Remy C., Bernardi T., Provot C., Tasse J., Sotto A., Lavigne J.-P. (2022). A Relevant Wound-Like in vitro Media to Study Bacterial Cooperation and Biofilm in Chronic Wounds. Front. Microbiol..

[18] Alves P.J., Barreto R.T., Barrois B.M., Gryson L.G., Meaume S., Monstrey S.J. (2021). Update on the role of antiseptics in the management of chronic wounds with critical colonisation and/or biofilm. Int. Wound J..

[19] Babalska Z.Ł., Korbecka-Paczkowska M., Karpiński T.M. (2021). Wound Antiseptics and European Guidelines for Antiseptic Application in Wound Treatment. Pharmaceuticals.

[20] O’Meara S., Al-Kurdi D., Ologun Y., Ovington L.G., James M.M.-S., Richardson R. (2014). Antibiotics and antiseptics for venous leg ulcers. Cochrane Database Syst. Rev..

[21] Malone M., Aljohani K., Jensen S., Gosbell I.B., Dickson H.G., McLennan S., Hu H., Vickery K. (2017). Effect of cadexomer iodine on the microbial load and diversity of chronic non-healing diabetic foot ulcers complicated by biofilm in vivo. J. Antimicrob. Chemother..

[22] Schwartz J.A., Lantis J.C., Gendics C., Fuller A.M., Payne W., Ochs D. (2012). A prospective, non comparative, multicenter study to investigate the effect of cadexomer iodine on bioburden load and other wound characteristics in diabetic foot ulcers. Int. Wound J..

[23] Bahamondez-Canas T.F., Heersema L.A., Smyth H.D.C. (2019). Current Status of In Vitro Models and Assays for Susceptibility Testing for Wound Biofilm Infections. Biomedicines.

[24] Guzmán-Soto I., McTiernan C., Gonzalez-Gomez M., Ross A., Gupta K., Suuronen E.J., Mah T.-F., Griffith M., Alarcon E.I. (2021). Mimicking biofilm formation and development: Recent progress in in vitro and in vivo biofilm models. IScience.

[25] Thaarup I.C., Bjarnsholt T. (2021). Current In Vitro Biofilm-Infected Chronic Wound Models for Developing New Treatment Possibilities. Adv. Wound Care.

[26] Pouget C., Dunyach-Remy C., Pantel A., Schuldiner S., Sotto A., Lavigne J.-P. (2021). New Adapted In Vitro Technology to Evaluate Biofilm Formation and Antibiotic Activity Using Live Imaging under Flow Conditions. Diagnostics.

[27] Freitas A.I., Vasconcelos C., Vilanova M., Cerca N. (2014). Optimization of an automatic counting system for the quantification of Staphylococcus epidermidis cells in biofilms. J. Basic Microbiol..

[28] Pouget C., Dunyach-Remy C., Pantel A., Boutet-Dubois A., Schuldiner S., Sotto A., Lavigne J.-P., Loubet P. (2021). Alternative Approaches for the Management of Diabetic Foot Ulcers. Front. Microbiol..

[29] Mirzahosseini H.K., Hadadi-Fishani M., Morshedi K., Khaledi A. (2020). Meta-Analysis of Biofilm Formation, Antibiotic Resistance Pattern, and Biofilm-Related Genes in *Pseudomonas aeruginosa* Isolated from Clinical Samples. Microb. Drug Resist..

[30] Ciofu O., Tolker-Nielsen T. (2019). Tolerance and Resistance of *Pseudomonas aeruginosa* Biofilms to Antimicrobial Agents—How *P. aeruginosa* Can Escape Antibiotics. Front. Microbiol..

[31] Estrela C.R.A., Estrela C., Reis C., Bammann L.L., Pécora J.D. (2003). Control of microorganisms in vitro by endodontic irrigants. Braz. Dent. J..

[32] Locker J., Fitzgerald P., Sharp D. (2014). Antibacterial validation of electrogenerated hypochlorite using carbon-based electrodes. Lett. Appl. Microbiol..

[33] Loose M., Naber K.G., Purcell L., Wirth M.P., Wagenlehner F.M.E. (2021). Anti-Biofilm Effect of Octenidine and Polyhexanide on Uropathogenic Biofilm-Producing Bacteria. Urol. Int..

[34] Kawamura M., Fujimura S., Tokuda K., Aoyagi T., Endo S., Kanamori H., Watanabe A., Kaku M. (2019). Mutant selection window of disinfectants for *Staphylococcus aureus* and *Pseudomonas aeruginosa*. J. Glob. Antimicrob. Resist..

[35] Gupta P., Bhatia M., Gupta P., Omar B.J. (2018). Emerging Biocide Resistance among Multidrug-Resistant Bacteria: Myth or Reality?. A Pilot Study. J. Pharm. Bioallied Sci..

[36] Torrico M., González N., Giménez M.J., Alou L., Sevillano D., Navarro D., Díaz-Antolín M.P., Larrosa N., Aguilar L., Garcia-Escribano N. (2010). Influence of Media and Testing Methodology on Susceptibility to Tigecycline of Enterobacteriaceae with Reported High Tigecycline MIC. J. Clin. Microbiol..

[37] Koburger T., Hübner N.-O., Braun M., Siebert J., Kramer A. (2010). Standardized comparison of antiseptic efficacy of triclosan, PVP-iodine, octenidine dihydrochloride, polyhexanide and chlorhexidine digluconate. J. Antimicrob. Chemother..

[38] Murray J.L., Kwon T., Marcotte E.M., Whiteley M. (2015). Intrinsic Antimicrobial Resistance Determinants in the Superbug *Pseudomonas aeruginosa*. mBio.

[39] Sa A., Sawatdee S., Phadoongsombut N., Buatong W., Nakpeng T., Sritharadol R., Srichana T. (2017). Quantitative analysis of povidone-iodine thin films by X-ray photoelectron spectroscopy and their physicochemical properties. Acta Pharm..

[40] Hirsch T., Koerber A., Jacobsen F., Dissemond J., Steinau H.-U., Gatermann S., Al-Benna S., Kesting M., Seipp H.-M., Steinstraesser L. (2010). Evaluation of Toxic Side Effects of Clinically Used Skin Antiseptics In Vitro. J. Surg. Res..

[41] López-Rojas R., Fernández-Cuenca F., Serrano-Rocha L., Pascual Á. (2017). In vitro activity of a polyhexanide-betaine solution against high-risk clones of multidrug-resistant nosocomial pathogens. Enferm. Infecc. Microbiol. Clin..

[42] Zhou Z., Wei D., Lu Y. (2015). Polyhexamethylene guanidine hydrochloride shows bactericidal advantages over chlorhexidine digluconate against ESKAPE bacteria. Biotechnol. Appl. Biochem..

[43] Garratt I., Aranega-Bou P., Sutton J.M., Moore G., Wand M.E. (2021). Long-Term Exposure to Octenidine in a Simulated Sink Trap Environment Results in Selection of *Pseudomonas aeruginosa*, Citrobacter, and Enterobacter Isolates with Mutations in Efflux Pump Regulators. Appl. Environ. Microbiol..

[44] Tasse J., Croisier D., Badel-Berchoux S., Chavanet P., Bernardi T., Provot C., Laurent F. (2016). Preliminary results of a new antibiotic susceptibility test against biofilm installation in device-associated infections: The Antibiofilmogram^®^. Pathog. Dis..

[45] Ferrer M.D., Rodriguez J.C., Álvarez L., Artacho A., Royo G., Mira A. (2017). Effect of antibiotics on biofilm inhibition and induction measured by real-time cell analysis. J. Appl. Microbiol..

[46] Haraszthy V.I., Reynolds H.S., Sreenivasan P.K., Subramanyam R., Cummins D., Zambon J.J. (2006). Media- and method-dependent variations in minimal inhibitory concentrations of antiplaque agents on oral bacteria. Lett. Appl. Microbiol..

[47] Durani P., Leaper D. (2008). Povidone–iodine: Use in hand disinfection, skin preparation and antiseptic irrigation. Int. Wound J..

[48] Fukuzaki S. (2006). Mechanisms of Actions of Sodium Hypochlorite in Cleaning and Disinfection Processes. Biocontrol Sci..

[49] Lefebvre E., Vighetto C., Di Martino P., Larreta Garde V., Seyer D. (2016). Synergistic antibiofilm efficacy of various commercial antiseptics, enzymes and EDTA: A study of *Pseudomonas aeruginosa* and *Staphylococcus aureus* biofilms. Int. J. Antimicrob. Agents.

[50] Tomic-Canic M., Burgess J.L., O’Neill K.E., Strbo N., Pastar I. (2020). Skin Microbiota and its Interplay with Wound Healing. Am. J. Clin. Dermatol..

[51] Kalan L.R., Meisel J.S., Loesche M., Horwinski J., Soaita I., Chen X., Uberoi A., Gardner S.E., Grice E.A. (2019). Strain- and Species-Level Variation in the Microbiome of Diabetic Wounds Is Associated with Clinical Outcomes and Therapeutic Efficacy. Cell Host Microbe.

[52] Pouget C. (2021). Modèles D’infection de la Plaie du Pied Chez le Diabétique: Approche In Vitro et In Vivo de la Formation de Biofilms de Bactéries Pathogènes Seules ou en Association. Ph.D. Thesis.

[53] Malanovic N., Ön A., Pabst G., Zellner A., Lohner K. (2020). Octenidine: Novel insights into the detailed killing mechanism of Gram-negative bacteria at a cellular and molecular level. Int. J. Antimicrob. Agents.

[54] Shepherd M.J., Moore G., Wand M.E., Sutton J.M., Bock L.J. (2018). *Pseudomonas aeruginosa* adapts to octenidine in the laboratory and a simulated clinical setting, leading to increased tolerance to chlorhexidine and other biocides. J. Hosp. Infect..

[55] Chadeau E., Dumas E., Adt I., Degraeve P., Noël C., Girodet C., Oulahal N. (2012). Assessment of the mode of action of polyhexamethylene biguanide against Listeria innocua by Fourier transformed infrared spectroscopy and fluorescence anisotropy analysis. Can. J. Microbiol..

[56] Andriessen A.E., Eberlein T. (2008). Assessment of a wound cleansing solution in the treatment of problem wounds. Wounds.

[57] Holloway B.W., Krishnapillai V., Morgan A.F. (1979). Chromosomal genetics of Pseudomonas. Microbiol. Rev..

[58] Watters C., Yuan T.T., Rumbaugh K.P. (2015). Beneficial and deleterious bacterial–host interactions in chronic wound pathophysiology. Chronic Wound Care Manag. Res..

[59] Monteiro-Soares M., Russell D., Boyko E.J., Jeffcoate W., Mills J.L., Morbach S., Game F., on behalf of the International Working Group on the Diabetic Foot (IWGDF) (2020). Guidelines on the classification of diabetic foot ulcers (IWGDF 2019). Diabetes/Metab. Res. Rev..

[60] Sotto A., Laurent F., Schuldiner S., Vouillarmet J., Corvec S., Bemer P., Boutoille D., Dunyach-Rémy C., Lavigne J.-P. (2021). Evaluation of the Use of Antibiofilmogram Technology in the Clinical Evolution of Foot Ulcers Infected by *Staphylococcus aureus* in Persons Living with Diabetes: A Pilot Study. J. Clin. Med..

[61] Olivares E., Badel-Berchoux S., Provot C., Prévost G., Bernardi T., Jehl F. (2020). Clinical Impact of Antibiotics for the Treatment of *Pseudomonas aeruginosa* Biofilm Infections. Front. Microbiol..

[62] Naudin B., Heins A., Pinhal S., Dé E., Nicol M. (2019). BioFlux^TM^ 200 Microfluidic System to Study A. baumannii Biofilm Formation in a Dynamic Mode of Growth. Methods Mol. Biol..

[63] Benoit M.R., Conant C.G., Ionescu-Zanetti C., Schwartz M., Matin A. (2010). New Device for High-Throughput Viability Screening of Flow Biofilms. Appl. Environ. Microbiol..

[64] Tremblay Y.D.N., Vogeleer P., Jacques M., Harel J. (2015). High-Throughput Microfluidic Method To Study Biofilm Formation and Host-Pathogen Interactions in Pathogenic Escherichia coli. Appl. Environ. Microbiol..

