# Supplementary material for: Antibiofilm Properties of Antiseptic Agents Used on Pseudomonas aeruginosa Isolated from Diabetic Foot Ulcers
Source: Int J Mol Sci. 2022 Sep 24;23(19):11270. doi: 10.3390/ijms231911270 (PMC9569737; doi:10.3390/ijms231911270)
Supplement: Supplementary file 1 [file ijms-23-11270-s001.zip › ijms-1874637-supplementary.pdf]

Supplementary material: Antibiofilm properties of antiseptics on *P.aeruginosa biofilm*

Table S1 : Bacterial load values of 72H biofilm for all strains

Strains	PAO1		PAC1		PAC2		PAC4	
Media	BHI	CWM	BHI	CWM	BHI	CWM	BHI	CWM
	175	124	158	218	97	141	93	208
	194	198	177	201	118	162	104	213
	207	383	121	286	125	254	179	297
	139	278	203	179	229	340	183	127
	118	244	119	233	117	310	129	254
	151	257	126	189	231	171	121	182
Mean	164	247.3333	150.6667	217.6667	152.8333	229.6667	134.8333	213.5

Table S2 : Biofilm percentage before and after 72h ATS contact in dynamic flow model

PAO1												
ATS	Sodium Hypochlorite				PHMB				Octenidine			
Media	BHI		CWM		BHI		CWM		BHI		CWM	
	DBD*	H	DBD	H	DBD	H	DBD	H	DBD	H	DBD	H
	14.36%	2.66%	29.71%	8.28%	6.51%	3.83%	8.88%	9.74%	7.33%	4.78%	8.55%	7.16%
	11.15%	4.19%	27.22%	14.96%	5.28%	5.56%	11.77%	8.14%	6.42%	4.63%	8.98%	14.60%
	11.65%	2.43%	33.83%	7.89%	6.85%	3.61%	7.89%	7.72%	6.71%	4.77%	7.50%	8.11%
	13.25%	3.85%	16.47%	10.31%	5.11%	5.31%	8.19%	6.47%	7.38%	4.30%	4.84%	4.23%
	7.13%	4.85%	4.53%	13.63%	23.91%	17.85%	10.46%	7.82%	11.24%	6.36%	13.31%	20.97%
	6.86%	5.84%	18.85%	7.10%	20.91%	12.71%	19.85%	7.62%	11.24%	4.16%	16.05%	10.38%
	5.52%	4.03%	11.80%	4.96%	4.89%	6.55%	11.37%	8.67%	39.93%	7.78%	12.49%	14.17%
	6.69%	6.07%	5.87%	6.88%	12.81%	10.94%	15.56%	6.11%	47.81%	16.35%	19.03%	22.59%
			3.27%	2.95%	23.91%	17.85%	10.90%	7.28%				
			6.85%	5.84%	20.91%	12.71%	5.13%	6.09%				
			7.28%	8.20%	4.89%	6.55%	9.65%	5.77%				
			7.71%	6.69%	12.81%	10.94%	17.33%	11.74%				
mean	9.04%	4.05%	11.06%	7.50%	10.14%	8.31%	10.73%	7.61%	12.41%	5.93%	10.44%	11.21%
P value	0.003906		0.02124		0.01135		0.002441		0.003906		0.8438	

PAC1												
ATS	Sodium Hypochlorite				PHMB				Octenidine			
Media	BHI		CWM		BHI		CWM		BHI		CWM	
	DBD	H	DBD	H	DBD	H	DBD	H	DBD	H	DBD	H
	16.46%	6.03%	11.22%	7.24%	18.18%	5.87%	4.55%	2.68%	15.83%	19.53%	6.39%	5.98%
	16.55%	6.09%	11.02%	9.03%	20.17%	4.80%	3.95%	2.53%	24.18%	19.76%	6.39%	5.87%
	21.10%	6.57%	10.09%	8.23%	18.81%	8.33%	3.97%	2.64%	17.56%	3.84%	4.86%	4.83%
	21.83%	4.80%	4.65%	3.52%	12.97%	5.20%	5.45%	4.70%	16.11%	7.74%	5.17%	5.01%
	5.45%	4.41%	14.46%	8.96%	18.10%	5.05%	6.75%	5.84%	19.86%	13.33%	8.36%	7.85%
	4.21%	4.07%	7.77%	6.44%	21.30%	5.96%	6.37%	6.76%	13.46%	6.76%	10.69%	10.05%
	4.07%	3.88%	7.21%	6.52%	24.81%	6.06%	5.69%	5.35%	22.15%	7.01%	9.48%	9.00%

	3.93%	2.67%	7.50%	4.17%	28.24%	7.19%	6.52%	3.68%	16.40%	6.25%	7.69%	7.36%
			15.97%	9.35%	23.91%	17.52%						
			12.57%	5.11%	20.91%	20.81%						
			14.54%	8.82%	25.47%	24.87%						
			14.54%	4.48%	22.30%	21.08%						
mean	9.07%	4.64%	8.78%	6.44%	19.85%	5.97%	5.30%	4.00%	17.89%	9.06%	7.13%	6.78%
P value	0.003906		0.0002441		0.0002441		0.01172		0.007813		0.003906	

PAC2

ATS	Sodium Hypochlorite				PHMB				Octenidine			
Media	BHI		CWM		BHI		CWM		BHI		CWM	
	DBD	H	DBD	H	DBD	H	DBD	H	DBD	H	DBD	H
	7.30%	4.02%	6.20%	3.89%	10.55%	5.14%	6.59%	5.91%	4.72%	4.16%	17.36%	3.61%
	5.80%	3.96%	4.54%	3.95%	12.31%	4.94%	6.83%	4.72%	6.42%	5.62%	6.35%	5.68%
	6.99%	4.55%	3.80%	3.35%	11.45%	4.75%	7.07%	6.22%	4.07%	3.73%	4.34%	4.31%
	9.56%	10.39%	5.95%	3.29%	8.67%	6.34%	9.53%	9.08%	6.29%	6.66%	4.02%	6.76%
	5.21%	4.41%	9.21%	5.26%	6.77%	2.65%	13.63%	9.26%	8.99%	8.43%	7.41%	8.07%
	4.91%	3.43%	10.45%	6.58%	28.41%	4.39%	7.07%	5.09%	10.38%	9.69%	12.00%	7.67%
	2.95%	2.42%	9.45%	7.56%	28.05%	6.50%	6.34%	6.22%	14.89%	14.19%	14.70%	9.13%
	3.07%	1.50%	10.21%	5.61%	26.38%	6.72%	7.24%	6.69%	20.58%	19.53%	11.46%	7.52%
					26.06%	20.93%	7.49%	3.42%	15.08%	12.95%		
						8.34%	9.23%	6.03%	24.87%	20.81%		
						8.25%	12.22%	6.77%	12.87%	16.17%		
						11.49%	7.64%	5.87%	25.61%	29.79%		
mean	5.34%	3.78%	7.02%	4.73%	15.37%	6.57%	8.16%	6.08%	10.88%	10.46%	8.56%	6.32%
P value	0.01953		0.003906		0.001953		0.0002441		0.1331		0.05469	

PAC4

ATS	Sodium Hypochlorite				PHMB				Octenidine			
Media	BHI		CWM		BHI		CWM		BHI		CWM	
	DBD	H	DBD	H	DBD	H	DBD	H	DBD	H	DBD	H
	16.49%	8.85%	16.55%	16.56%	15.99%	5.74%	20.18%	12%	7.45%	3.68%	22.84%	10.36%
	17.18%	6.35%	23.28%	14.29%	16.92%	5.37%	13.82%	11%	6.26%	4.04%	35.59%	11.25%
	28.57%	8.29%	21.62%	18.64%	13.35%	5.79%	23.84%	5%	7.50%	3.99%	21.49%	12.75%
	25.80%	7.06%	17.32%	15.96%	17.78%	6.29%	19.42%	19%	7.45%	3.69%	15.97%	14.21%
	20.16%	13.61%	7.46%	3.30%	9.57%	9.83%	12.60%	6%	12.93%	9.83%	20.01%	10.08%
	27.65%	10.75%	9.50%	2.56%	8.37%	6.81%	18.95%	10%	19.41%	8.12%	15.86%	12.15%
	23.22%	17.46%	12.75%	2.18%	7.50%	6.53%	15.84%	18%	10.49%	5.14%	12.86%	10.67%
	16.60%	10.84%	7.14%	2.72%	7.45%	3.34%	15.37%	12%	7.77%	6.67%	15.44%	7.94%
					11.24%	10.37%	43.44%	43%				
					11.24%	11.01%	58.29%	58%				
mean	21.45%	9.89%	13.22%	6.59%	14.24%	9.17%	22.75%	16.47%	9.24%	5.27%	19.08%	11.03%
P value	0.003906		0.007813		0.001709		0.006104		0.003906		0.003906	

*DBD= débridement = before antiseptic contact

Table S3. Studies assessing Minimum Inhibitory Concentrations (MIC) of sodium hypochlorite, PVPI, PHMB and octenidine.

Antiseptics	MIC	Dilution	*Pseudomonas* strains	Method	References
Sodium Hypochlorite	1%	1:5	ATCC 27853	Personal	[31]
	0.025%	1:20	NCIMB 12469	Personal	[32]
	800 $mg.L^{-1}$	1:6	ATCC27853	Microdilution broth	[33]
	512 $mg.L^{-1}$	1:10	60 clinical isolates	Microdilution broth	[34]
	400 $mg.L^{-1}$	1:12	Clinical isolate	Microdilution broth	[33]
	312.5 $mg.L^{-1}$	1:16	ATCC 27853	Microdilution broth	[35]
	312.5 $mg.L^{-1}$	1:16	10 clinical isolates	Microdilution broth	[35]
	1,666 $mg.L^{-1}$	1:3	PAO1 and 3 clinical isolates	Microdilution broth	Current study
PVPI	1,024 $mg.L^{-1}$	1:100	ATCC 15442	Microdilution broth	[37]
	5%	1:2	UCBPP-PA14	Personal	[38]
	0.04%	1:250	ATCC 15442	Microdilution broth	[39]
	7,500 $mg.L^{-1}$	1:16	ATCC 27853	Microdilution broth	[40]
	12,500 $mg.L^{-1}$	1:8	PAO1 and 3 clinical isolates	Microdilution broth	Current study
PHMB	2 $mg.L^{-1}$	1:512	ATCC 15442	Microdilution broth	[37]
	4 $mg.L^{-1}$	1:256	ATCC 27853	Microdilution broth	[41]
	8 $mg.L^{-1}$	1:258	3 clinical isolates	Microdilution broth	[41]
	16-32 $mg.L^{-1}$	1:64 to 1:32	25 clinical isolates	Microdilution broth	[42]
	7.8 $mg.L^{-1}$	1:128	ATCC 27853	Microdilution broth	[33]
	15.6 $mg.L^{-1}$	1:64	Clinical isolates	Microdilution broth	[33]
	10 $mg.L^{-1}$	1:100	ATCC 27853	Microdilution broth	[40]
	15.6 $mg.L^{-1}$	1:64	PAO1 and 3 clinical isolates	Microdilution broth	Current study
Octenidine	2 $mg.L^{-1}$	1:256	ATCC 15442	Microdilution broth	[37]
	4-16 $mg.L^{-1}$	1:128-1:32	2 clinical isolates	Microdilution broth	[43]
	3.9 $mg.L^{-1}$	1:128	ATCC 27853	Microdilution broth	[33]
	3.9 $mg.L^{-1}$	1:32	Clinical isolate	Microdilution broth	[33]
	1 $mg.L^{-1}$	1:500	ATCC 27853	Microdilution broth	[40]
	7.8 $mg.L^{-1}$	1:64	PAO1 and 3 clinical isolates	Microdilution broth	Current study
